# Studies of Interaction Mechanism between Pyrido [3,4-*d*] Pyrimidine Inhibitors and Mps1

**DOI:** 10.3390/molecules26165075

**Published:** 2021-08-21

**Authors:** Cheng Xing, Xiaoping Zhou, Chengjuan Chen, Wei Sun, Qingchuan Zheng, Di Liang

**Affiliations:** 1 School of Pharmaceutical Sciences, Jilin University, Changchun 130021, China; xingcheng18@mails.jlu.edu.cn (C.X.); zhouxp@jlu.edu.cn (X.Z.); chencj18@mails.jlu.edu.cn (C.C.); wsun@jlu.edu.cn (W.S.); 2 Laboratory of Theoretical and Computational Chemistry, Jilin University, Changchun 130023, China; zhengqc@jlu.edu.cn

**Keywords:** Monopolar spindle 1, pyrido [3,4-*d*] pyrimidine, molecular docking, molecular dynamics simulations, drug design

## Abstract

Monopolar spindle 1 (Mps1), a dual-specific kinase, is related to the proper execution of chromosome biorientation and mitotic checkpoint signaling. The overexpression of Mps1 promotes the occurrence of cancer or the survival of aneuploid cancer cells, in other words, the reduction of Mps1 will severely reduce the viability of human cancer cells. Therefore, Mps1 is a potential target for cancer treatment. Recently, a series of novel pyrido [3,4-*d*] pyrimidine derivatives targeting Mps1 with high biological activity were synthesized. The crystal structure of Mps1 in complex with pyrido [3,4-*d*] pyrimidine derivatives was also reported, but there were no specific mechanism studies for this series of small molecule inhibitors. In this study, complexes binding modes were probed by molecular docking and further validated by molecular dynamics simulations and the molecular mechanics/generalized Born surface area (MM/GBSA) method. The results indicated that the van der Waals interactions and the nonpolar solvation energies were responsible to the basis for favorable binding free energies, all inhibitors interacted with residues I531, V539, M602, C604, N606, I607, L654, I663, and P673 of Mps1. By analyzing the hydrogen bonds, we found the residues G605 and K529 in Mps1 formed stable hydrogen bonds with compounds, it was more conducive to activities of Mps1 inhibitors. According to the above analysis, we further designed five new compounds. We found that compounds IV and V were better potential Mps1 inhibitors through docking and ADMET prediction. The obtained new insights not only were helpful in understanding the binding mode of inhibitors in Mps1, but also provided important references for further rational design of Mps1 inhibitors.

## 1. Introduction

The spindle assembly checkpoint (SAC) is a major cell cycle control mechanism that guards against chromosome missegregation and the subsequent production of aneuploid daughter cells. Most cancer cells are aneuploid and frequently missegregate chromosomes during mitosis. Indeed, aneuploidy is a common characteristic of tumors. Therefore, tumorigenesis depends on aneuploidy and chromosomal instability [1]. Monopolar spindle 1 (Mps1), as the core component of SAC, can ensure proper biorientation of sister chromatids on the mitotic spindle at kinetochores [2]. Moreover, Mps1 is hardly expressed in normal cells, but highly expressed in cancer cells, and cancer cells rely heavily on Mps1 to cope with aneuploidy caused by abnormal chromosome numbers [3,4]. Inhibition of highly expressed Mps1 can reduce the survival rate of tumor cells [5,6,7]. In recent studies, it can be found that Mps1 depletion or inhibition can effectively kill tetraploid cancer cells, by assessing the ploidy state of tumor response to Mps1 inhibition [8]. In addition, the latest studies have found synergistic effects between Mps1 inhibitors and antimitotic agents or radiotherapy [9,10,11]. In summary, Mps1 is considered to be one of the most promising treatment targets for cancer.

According to different binding modes with Mps1 kinase, the reported Mps1 inhibitors can be divided into two types [12]. Type I Mps1 inhibitors interact with the ATP-binding sites in the phosphorylated Mps1 kinase conformation [13,14,15], Type II Mps1 inhibitors are the combination of inhibitors with other pockets of the kinase, these inhibitors indirectly block ATP binding by changing the conformation of Mps1 kinase [16,17]. Most Mps1 inhibitors are type I inhibitors, such as MPI-0479605 [18], TC Mps1 12 [19], BOS172722 [20]. Because Mps1 has many highly homologous proteins such as Aurora, CDK2, GSK3β, etc. [21], selectivity is a key factor for the design of novel Mps1 inhibitors.

**Figure 1:**
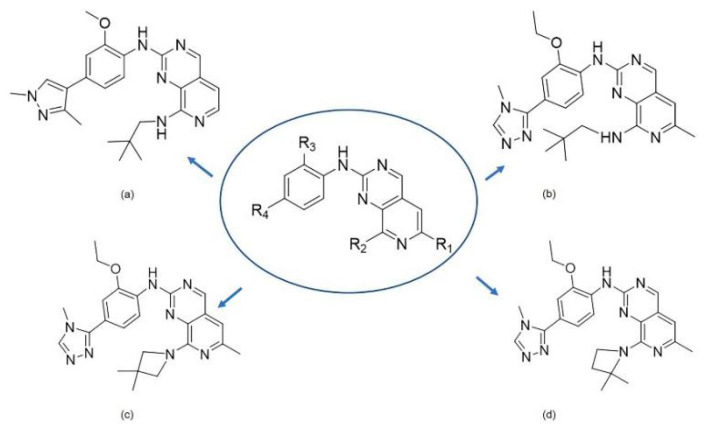
The chemical structures of (**a**): 14, (**b**): BOS172722, (**c**): 47, (**d**): 48.

Recently, a series of Mps1 inhibitors with a backbone of pyrido [3,4-*d*] pyrimidines have been discovered, which showed excellent potency and kinase selectivity [20,21]. One of the inhibitors, BOS172722, has entered the first clinical stage. It is a highly potent, selective, and orally bioavailable inhibitor of Mps1. The current target indication for BOS172722 is triple-negative breast cancer, and the synergy observed with paclitaxel [22]. Although crystal structures of Mps1 in complex with pyrido [3,4-*d*] pyrimidine derivatives are reported [20], the binding modes of such compounds with Mps1 have not been investigated in detail. Thus, it is necessary to study the specific binding mode between these inhibitors and Mps1. In this study, comprehensive simulation methods were used to clarify the modes of the binding between Mps1 and pyrido [3,4-*d*] pyrimidine inhibitors. First, the modeling of Mps1 structure was performed. The molecular dynamics simulation was performed in the Amber program to optimize the modeled structure. Second, we selected 26 pyrido [3,4-*d*] pyrimidine inhibitors from the same laboratory and connected the inhibitors with Mps1 by the Autodock software. We found some differences between compounds 14 and BOS172722 were the substituents at the R_4_ position—pyrazole and triazole, respectively, but the literature did not specify the effect of different R_4_ substituents on inhibitor activity; the substituents at the R_1_ position are with or without methyl. According to the literature, the 6-methyl group, which is important for CDK2 selectivity and for the reduction in HLM metabolism, is located close to the side chain of the “gatekeeper” residue Met602, and the ethoxy moiety also important for selectivity binds in the selectivity pocket above the hinge [20,21]. In addition, the activities of compounds 47 and 48 differ by several tens of times, but they are only isomers in the R_2_ substituent, implicating the shape of R_2_ substituent was also relatively important on the activity. Finally, four compounds (Figure 1) were selected and subjected to molecular dynamic (MD) simulations, and the protein-ligand binding modes were identified by per-residue energy decomposition and hydrophobic effect analysis.

## 2. Results and Discussion

### 2.1. Mps1 Modeling Verification

The crystal structure of the Mps1 protein (PDB ID:6H3K) was downloaded from the PDB database. We used MODELLER 9.13v software package [23] to complete the missing residues of the protein, and the modeled structure of Mps1 (PDB ID: 6H3K) was evaluated with PROCHECK, which showed the most consistent results with the Ramachandran plot. In total, 225 residues were found in most favored regions (~90.9%), 19 residues were in additional allowed regions (~7.5%), and 4 residues were in disallowed regions (~1.6%), which proved that the comparative modeling of the three-dimensional (3D) structure of Mps1 using knowledge-based computational approaches was fairly successful (Figure S1). In summary, the 3D structure of Mps1 modeled based on the homology modeling method was reasonable.

**Figure 2:**
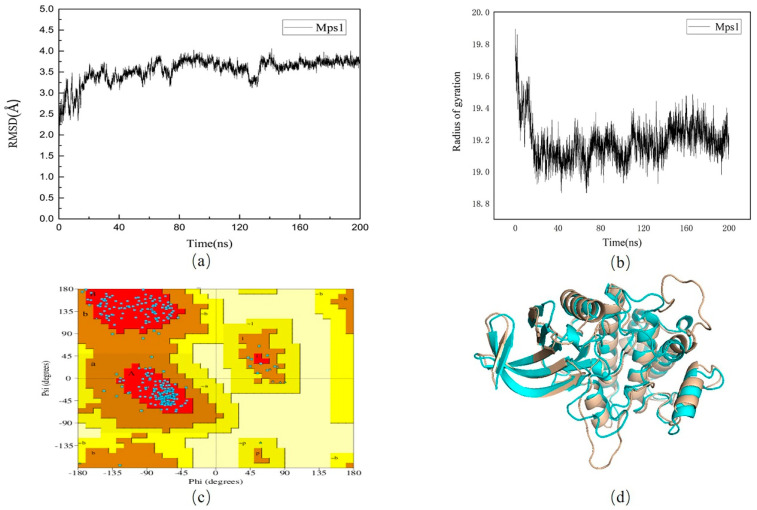
(**a**): Root mean square deviation (RMSD) values of 6H3K protein in apo protein; (**b**): The radius of gyration of 6H3K protein in apo protein. (**c**): Ramachandran diagram of Mps1 protein structure after optimization. (**d**): Comparison of conformational overlap between the original 6H3K protein (wheat) and the 200 ns protein (blue).

To obtain a stable conformation, we performed a 200 ns molecular dynamic simulation on the protein without small molecules. As shown in Figure 2a, there were some fluctuations in the entire system at the early stage of the trajectory and the root mean square deviation (RMSD) of the protein fluctuates within 1 Å after 140 ns, indicating that the system has stabilized. In Figure 2b, we found radius of gyration (Rg) was decreasing. The Rg was usually used for evaluating the compactness of protein structure. Therefore, the conformation of the protein was more and more stable. A total of 500 frames were extracted from 40 ns after stabilization to generate a stable conformation of the protein. The Ramachandran quality assessment results showed (Figure 2c) that the amino acid residues originally derived from the disallowed region and the loosely permitted region have been optimized. In Figure 2d structure superposition, the difference between the two structures could be observed intuitively.

### 2.2. Molecular Docking

#### 2.2.1. Validation of Docking Protocol

**Figure 3:**
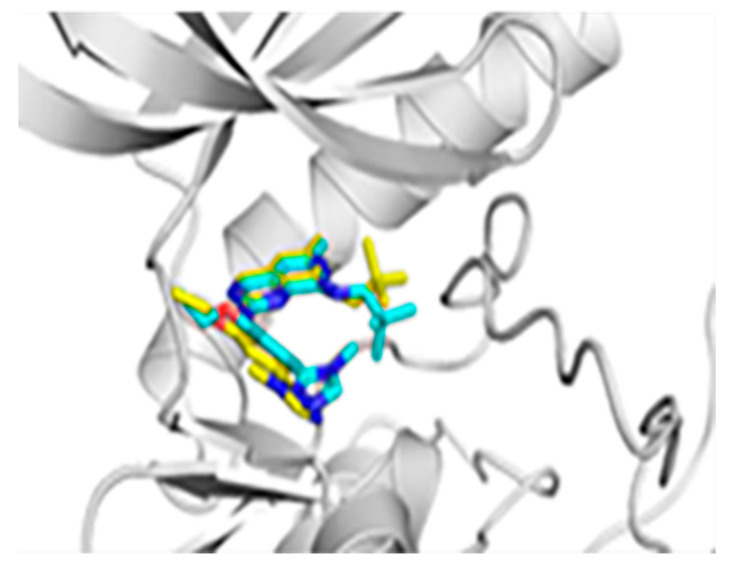
Redocking of BOS172722. The green stick stands for the docked pose, while the yellow represents the original conformation.

In this article, the small molecule BOS172722 in the original PDB file with the stabilized and repaired protein was redocked, and then the crystal structure and the RMSD value of the docking result were analyzed through VMD. The result showed that the ligand in the molecular docking result and the RMSD value between the binding site and original pose was 0.49 Å, which was less than 1 Å. The result was shown in Figure 3, the docked ligand and the original ligand were almost superimposed.

We found that the order of the binding energy in the compounds obtained by docking and the experimental value of IC_50_ was consistent as listed in Table 1. This showed that the molecular docking method and the conformational selection criteria used in this article was reliable and had the ability to select reasonable conformations, and the best selected conformation could be used for further studies.

#### 2.2.2. Comparison of the General Structure of Different Pyrido [3,4-*d*] Pyrimidine Inhibitors

It can also be noticed that the order of binding energy, which was consistent with the ranking of the experimental IC_50_ values [20,21], and the number of selected cluster conformations was the highest. This showed that the molecular docking method and conformation selection criteria used in this article were very reliable and had the ability to generate reasonable conformations. The best conformation chosen could be exploited in the following study.

According to the molecular docking of the compound, we found that the backbone of the pyrido [3,4-*d*] pyrimidine compound and the hinge region of Mps1 had a strong hydrophobic effect, which also explains why this type of compound has strong activity. Additionally, we observed that the pyrimidine ring of the compounds formed a hydrogen bond with Gly605. Therefore, Gly605 was regarded as an important residue of Mps1. By docking compounds with different structures, we found that the R_5_ groups of imidazole and triazole could form hydrogen bonds with Val529 of Mps1, while those of pyrazoles could not. It provided a theoretical basis for the future design in such compounds. In addition, neopentyl chains at the R_2_ position could bind tightly in this pocket. Further research has shown that only chains similar in shape to neopentyl chains can be incorporated in this pocket. Therefore, the shape of the R_2_ group of a substituent exhibits a greater influence on the activity of a compound. We found that the methyl group at the R_1_ position was also important, because it could bind closer to the “gatekeeper” residue Met602 of Mps1, thereby allowing the compound to bind with the protein more tightly.

**Figure 4:**
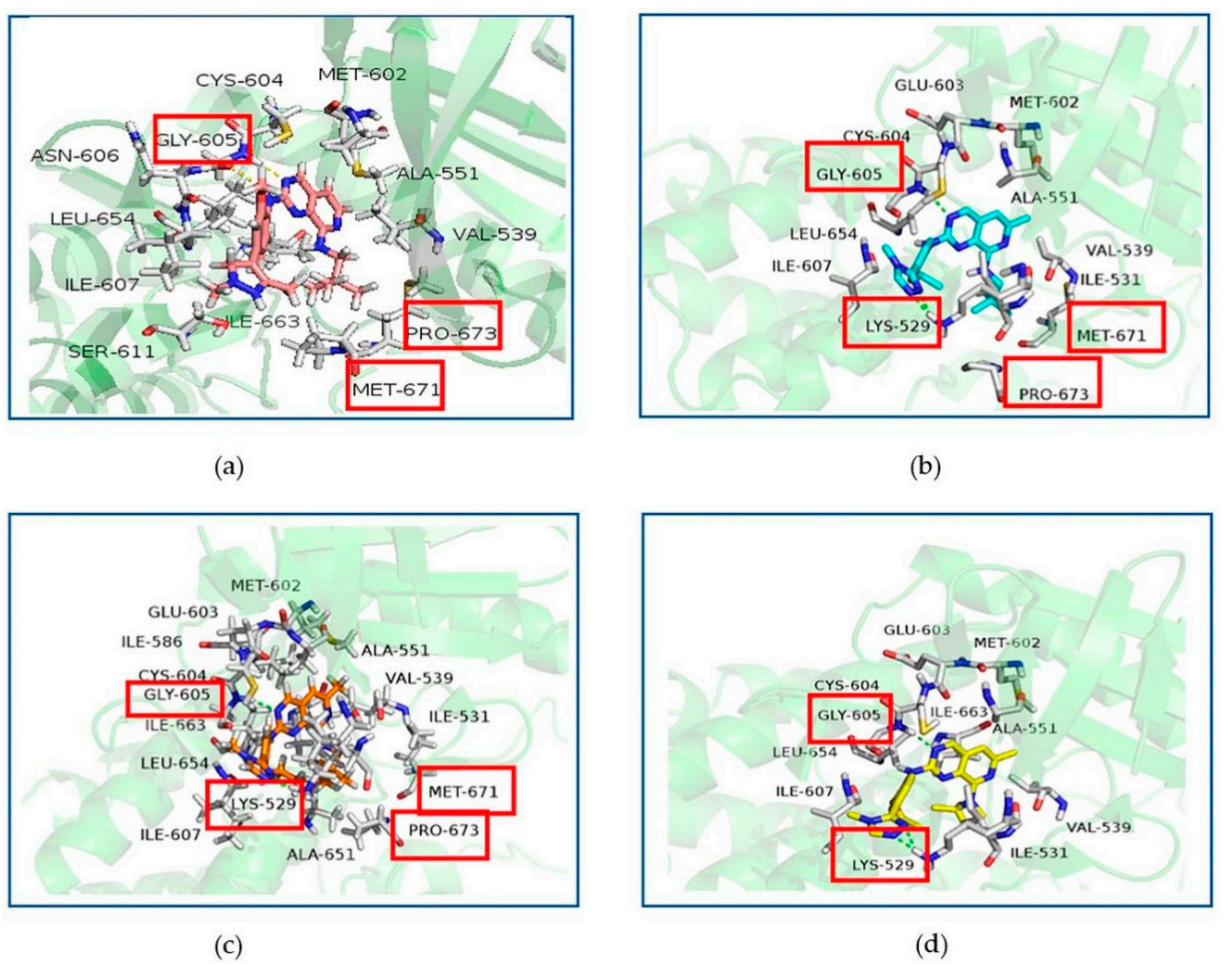
Docking result of compounds with Mps1. Interactions of inhibitors residues with hydrogen bonds in yellow (**a**) and green (**b**–**d**) dotted line. (**a**): compound A, (**b**): compound BOS172722, (**c**): compound C, (**d**): compound D.

Figure 4 showed the binding patterns between Mps1 and representative small molecules. Figure 4a showed the binding pattern between Mps1 and compound 14 (A), with the yellow dashed lines representing the hydrogen bond formed. In Figure 4b–d, the green dashed lines represent the hydrogen bonds formed between Mps1 and compounds 36 (BOS172722), 47 (C), and 48 (D), respectively.

According to our analysis, all the four compounds could form hydrogen bonds with Gly605. Therefore, Gly605 was regarded as an important amino acid residue for Mps1. In addition, all the four compounds formed hydrophobic interactions with residues Val539, Ala551, Glu603, Cys604, Ile607, and Leu654, suggesting that these residues were important for the binding of compounds with Mps1. There were also differences between the binding patterns of compounds A, BOS172722, C, and D. Specifically, compounds BOS172722, C, and D formed hydrogen bonds with Lys529 of Mps1, whereas compound A did not, which may explain the poor activity of compound A. Additionally, residues Met671-Pro673 of Mps1 formed a hydrophobic pocket that wrapped compounds A, BOS172722, and C without compound D. Thus, it explained the reason for the poor activity of compound D. The selected docked pose of the most representative compounds A, BOS172722, C, and D were taken as an initial structure to perform molecular dynamics simulation.

### 2.3. Molecular Dynamics Simulation

Molecular dynamics simulation could provide detailed information about structural changes at the atomic level, and has been widely used in the structural study of protein molecules. To better understand the stability and interaction under the conditions of the surrounding environment, a 200 ns molecular dynamics simulation was performed of four complexes using Amber software [24]. The structural changes and dynamic behavior of protein–ligand complex was analyzed through root mean square deviation (RMSD), root mean square fluctuation (RMSF), the molecular mechanics/generalized Born surface area (MM/GBSA) method, and hydrogen-bond variations.

**Figure 5:**
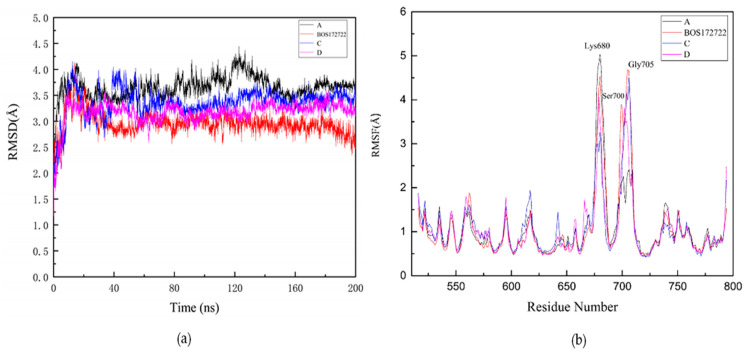
Stability of ligands in protein-ligands during 200 ns molecular dynamics (MD) simulation. (**a**) Root mean square deviation (RMSD) values of protein–A complex (black); protein–BOS172722 complex (blue); protein–C complex (purple); protein–D complex (red). (**b**) Root mean square fluctuation (RMSF) values of protein–A complex (black); protein–BOS172722 complex (blue); protein–C complex (purple); protein–D complex (red) during 200 ns MD simulations.

The RMSD of the protein backbone atoms of four complexes were calculated to evaluate the stability of the system. As shown in Figure 5a, it is obvious that the RMSD values of the four complexes are all convergent, and the system maintains equilibrium during the last 60 ns. In addition, the RMSD of the four complexes were lower than the protein, with the average values of 3.55 ± 0.22 Å, 2.76 ± 0.22 Å, 2.86 ± 0.27 Å, 2.97 ± 0.22 Å for A, BOS172722, C, and D, respectively. This observation might be attributed to the binding of the ligand that helps stabilize the structure of the protein. Therefore, RMSD provided a suitable basis for further research and all subsequent analysis of four complexes were performed on the last 60 ns of the simulation trajectories.

The RMSF was an analysis method that can measure the fluctuation of each amino acid residue in the dynamic state of a protein during an MD simulation. A higher RMSF value indicates a residue has low stability; conversely, a lower RMSF value indicated that a residue has high stability. In order to evaluate the influence of different small molecule inhibitors on the fluctuation of each amino acid residue in the protein during the MD simulations, we calculated the RMSF values of each amino acid residue in the four systems. The results were shown in Figure 5b. The RMSF profiles of each residue in the four systems showed similar patterns, all of which were consistent with that of the apo protein. We also found that three residues, namely Lys680, Ser700, and Gly705, which were far away from the binding site, exhibited relatively high RMSF values, probably because these residues were located in the loop region of the kinase, which has greater structural flexibility. We also found that Pro673 had a higher RMSF value in compound D than in compounds A, BOS172722, and C.

### 2.4. Calculation of Binding Free Energy Using MM/GBSA

#### 2.4.1. Binding Free Energy Calculations

To quantitatively gain insight into different contributions to the affinity of ligands binding to Mps1 and the main driving force for their binding, the MM/GBSA method was used to calculate binding free energy for each complex [25]. The binding free energies were calculated based on 500 frames uniformly selected from at the last 10 ns post-kinetic trajectory, and then their average values were calculated. The binding free energies were divided into electrostatic energy, van der Waals interaction energy, non-polar desolvation effect, and polar desolvation effect. We also calculated the conformational entropy. The results were listed in Table 2. The binding free energies of these four systems were in the order of A (-29.07+/-3.37 kcal/mol) < D (-35.11+/-3.25 kcal/mol) < BOS172722 (−38.46 +/ −2.97 kcal/mol) < C (−39.95+/−3.52 kcal/mol). In other words, the binding between Mps1 and compound A was weaker than those between Mps1 and the other three compounds, a conclusion that was consistent with the experimental results.

In all the four systems, the non-polar interaction term (∆G_nonp_) was the dominant type of energy contribution. ∆G_nonp_ was the sum of ∆E_vdW_ and ∆G_SA_ and mainly came from van der Waals interactions. The ∆G_pol_ values for all the systems were positive, which also indicated that polar interactions were not favorable for the bindings between Mps1 and its inhibitors. The entropy changes (−TΔS) were positive, indicating that the bindings between Mps1 and its inhibitors were somewhat curtailed, and we found that the binding processes were mainly enthalpy-driven with minimal influence from entropy. In general, van der Waals interactions and nonpolar solvation energies were the basis for favorable binding free energies, suggesting that the shape complementarity between Mps1 and its inhibitors was also important for their bindings.

#### 2.4.2. Binding Free Energy Decomposition

**Figure 6:**
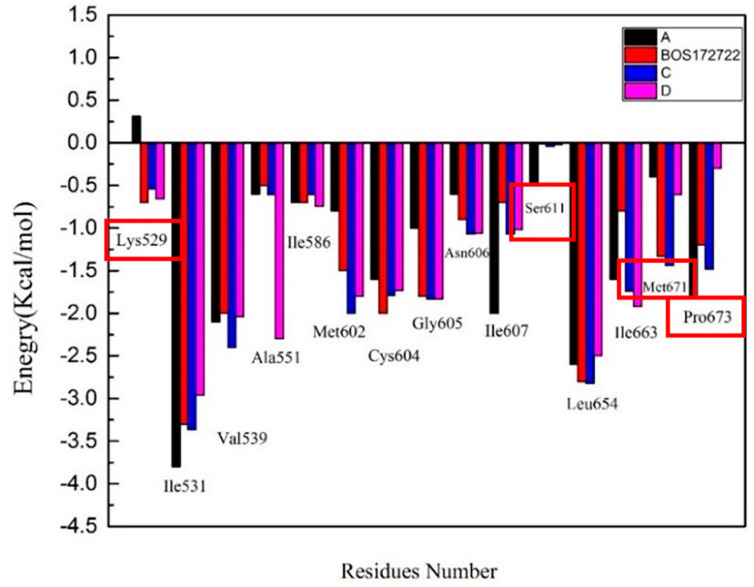
The column charts depicting the contributions of each residue to the total binding free energy.

To further understand the mechanism underlying the interactions between Mps1 and the compounds, the binding free energy was decomposed into inhibitor-residue pairs to obtain more detailed insight into the interaction mechanism between Mps1 and the compounds. If the interaction energy between a residue and a compound was lower than −0.5 kcal/mol, the residue could be regarded as essential for ligand binding. The results were displayed in Figure 6 and Table S1.

Based on these data, we found that the major favorable energy contributions in the four complexes originate from residues Ile531, Val539, Cys604, Gly605, Asn606, Ile607, Leu654, and Ile663, whose contribution to ΔG_bind_ were lower than −0.50 kcal/mol. All these residues, except Gly605, had a clear van der Waals interactions contribution. Therefore, hydrophobic interactions should be the main binding mode. In the Mps1-BOS172722, Mps1-C, and Mps1-D complexes, we detected a strong contribution from Lys529 as well with binding energies of −0.7 kcal/mol, −0.54 kcal/mol, −0.66 kcal/mol, respectively; whereas in the Mps1-A complex, the binding free energy of this residue was positive. This was also the reason for the poor activity of inhibitor A. The difference in structure between compound C and compound D was mainly that the substituent at the R_2_ position were isomers. Through the previous molecular docking, it was found that the R_2_ substituent of compound C could enter the hydrophobic pocket formed by Met671-Pro673. However, the R_2_ substituent of compound D could not enter the hydrophobic pocket due to steric hindrance. Experiments had also found that the binding free energies of residues Met671 and Pro673 in the compound C (−1.5 kcal/mol and −1.7 kcal/mol, respectively) were much higher than in the compound D (−0.6 kcal/mol and −0.3 kcal/mol, respectively). This also further verified that the substituents at the R_2_ position of the compound did not form a strong interaction with residues Met671 and Pro673. The correctness of the results obtained from the docking was also verified. Finally, we found that the binding free energy of Ser611 was -0.5 kcal/mol in complex A, while it was < −0.1 kcal/mol in the other three complexes. It was likely due to a methyl group at position 3 of the Pyrazole ring that could have a stronger hydrophobic interaction with the protein. The other three complexes did not have corresponding substituents that can bind to residue Ser611.

### 2.5. Binding Modes between Mps1 and Its Inhibitors

Non-covalent interactions (e.g., hydrogen bonds, hydrophobic interactions, salt bridges, etc.) play important roles in stabilizing protein conformations, realizing protein biological functions, and facilitating interactions between protein and other molecules. Table 3 showed the occupancy of hydrogen bonds between ligand and the protein in the four systems.

According to our analysis, four inhibitors formed a stable hydrogen bond with Gly605 of the protein, indicating that this hydrogen bond had great contribution to the substrate binding. Previous studies had similar conclusion that the interaction between the inhibitor and Gly605 was indispensable in Mps1 inhibitors [20]. It was worth noting that the triazole ring in the structure of the other three compounds could form stable hydrogen bonds with Lys529, which made the ligand stronger binding to the protein. It was also reasonable to explain our previous computation results that complex B, complex C, and complex D showed the binding free energy and electrostatic energy were stronger than complex A (Table 2). In addition, we decomposed the binding free energy to each residue. The results showed that both Lys529 and Gly605 contributed significantly to the inhibitors binding in Mps1. Accordingly, we concluded that apart from the hydrogen bond between Gly605 and compound, the hydrogen bond between Lys529 and compound was also the key to stable inhibitor binding to the protein.

**Figure 7:**
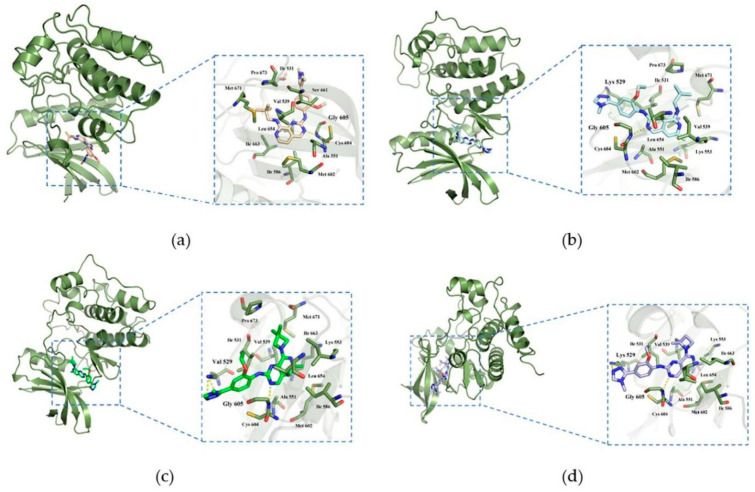
Complex binding modes. (**a**): Mps1-A; (**b**): Mps1-Bos 172722; (**c**): Mps1-C; (**d**): Mps1-D.

In order to elucidate the binding patterns of Mps1 with the four compounds, 500 frames were extracted from the last 10 ns of the MD simulations to generate the structures representing each of the complexes. The interaction patterns of these complexes were shown in Figure 7. The important residues were marked and highlighted in Figure 7, and the yellow dashed lines are the hydrogen bonds formed between the compounds and the proteins. The diagram showed that all the small molecules formed hydrogen bonds with Gly605 of Mps1, which is a common feature of most Mps1 inhibitors. We found that residues Cys604-Ile607 formed a hydrophobic pocket, which enhanced the hydrophobic effect and thus allowed the ligand to bind more stably to the protein.

The pyrimidine ring of compound A formed a hydrogen bond with Gly605, which was represented as the main hydrogen bonding interaction between Mps1 and compound A. From the decomposition of the free energy contributed by each amino acid indicated binding, we found that the contribution of Ser611 in system A was much more than those in the other systems. The hydrophobic pocket formed the activation ring consisting of residues Met671-Pro673, because the 6-methyl-N^8^-neopentyl group of compound A was tightly bound in this hydrophobic pocket in the Mps1-A complex.

Systems BOS172722 and C shared a common feature: the triazole ring at the R_5_ position of compounds BOS172722 and C formed hydrogen bonds with Lys529, an observation consistent with the molecular docking analysis (which showed that both triazole and imidazole could potentially form hydrogen bonds with Lys529), but this was not found in previous crystal structures. This may be due to the fact that a crystal structure only captures the conformation of a protein at a specific time point (in this case, a time point at which the hydrogen bonds were not formed), while molecular dynamics simulations can describe conformation changes of a protein during a period of time. We also found that the hydrophobic interaction binding pocket mentioned above was also found present in complexes Mps1-BOS172722 and Mps1-C. This was probably because the neopentyl chains of compounds A, BOS172722, and C have similar shapes, thereby leading to the formation of similar hydrophobic contacts. There was an additional methyl group at the R_1_ position in compounds BOS1722 and C. It has also been reported that 6-methylated compounds demonstrated a significant improvement in selectivity for Mps1 over CDK2 [20]. Selectivity plays an important role. Therefore, addition of a methyl group to the pyridine ring of BOS172722 will be essential. We also found that compounds BOS172722, C, and D formed pi-alkyl interactions with Lys533, while compound A did not. The hydrogen bonding interaction described above was observed in system D, but the 2,2-dimethylazetidine at the R_2_ position of compound D did not form hydrophobic contacts with residues Met671-Pro673. Compounds C and D were hundreds of times more active simply because the two methyl substitution positions were different. Therefore, it suggested that the shape of the compound at the R_2_ position may be more important, and more attention should be paid to the shape of the R_2_ position when designing novel Mps1 inhibitors.

### 2.6. Hydrophobic Interaction Analysis

**Figure 8:**
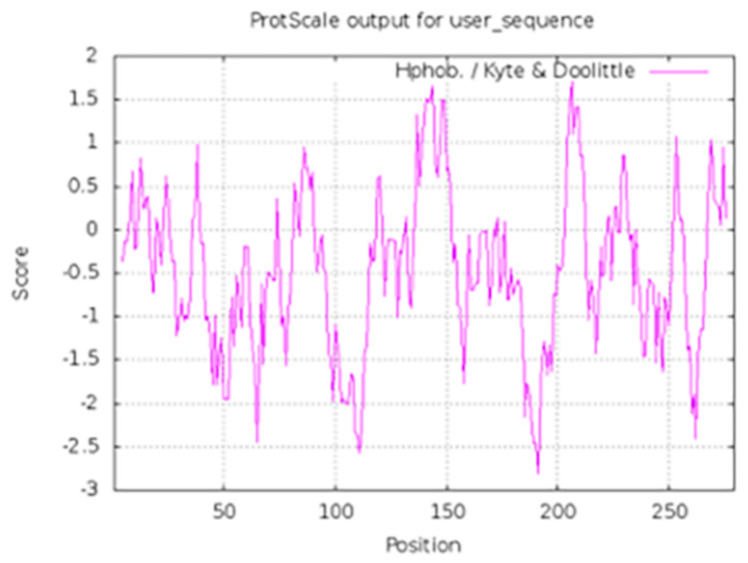
Hydrophobic interaction analysis of Mps1-BOS172722. The value 1 in the abscissa of the figure was residue 515.

In the case of hydrophobic effect, we used ExPASy Server [25], and the common hydrophobic effect of this small molecule compound has been studied in the clinical 1 inhibitor BOS172722. The results are shown in Figure 8 and positive value represents hydrophobicity (higher values mean stronger hydrophobicity). We found that there was a total of ten residues that formed strong hydrophobic interaction with the inhibitor and seven of them were key residues. They were Ile531, Val539, Cys604, Gly605, Leu654, Ile663, Pro673, and all located in the ATP binding pocket. This was consistent with the results obtained by molecular docking and dynamics simulations.

### 2.7. Drug Design

**Figure 9:**
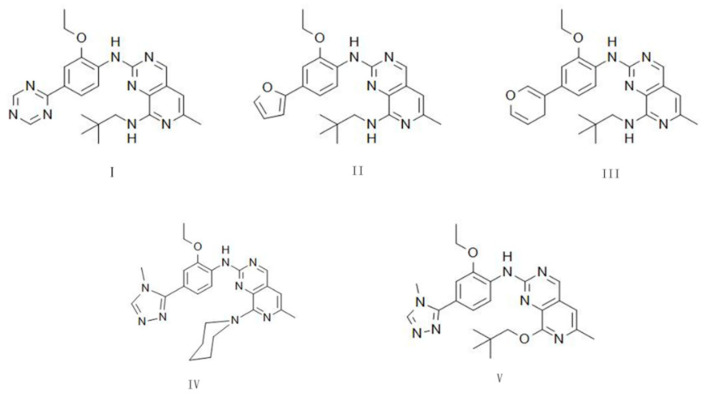
The chemical structures of new compound.

Based on the above results, we found that the pyrido [3,4-*d*] pyrimidine skeleton has a close hydrophobic interaction with the hinge region of Mps1, so we used this skeleton as the core structure. Because BOS172722 can form stable hydrogen with Lys529 bond and had stable hydrophobic interactions with key hydrophobic pockets. Considering these, the core fragment of BOS172722 was chosen to act as the starting structure for new inhibitor design. According to the LUDI-guided identification of interactions fragments, five new compounds were obtained and structures were shown in Figure 9. We used density functional theory B3LYP/6-G in Gaussian09 software to optimize the molecular geometry of five small molecules. We used Autodock for docking, it can be noticed that the binding energy of I, IV, and V has higher values than that of II, III.

In development of a new drug, the pharmacokinetic prediction for absorption, distribution, metabolism, and excretion (ADME) of a drug were necessary. Additionally, some toxicological parameters (Tox) also need to be evaluated. In this work, several parameters of the designed compounds, such as molecular weight (MW), octanol/water partition coefficient (cLogP), hydrogen bond acceptor count (HBA), hydrogen bond donor count (HBD), topological polar surface area (TPSA), and rotatable bond count (RB), were determined. These parameters were then subjected to toxicological evaluation compared based on the Lipinski, Veber, and Pfizer rules. Our reference ligand meets all the parameters of the Lipinski as shown in Table 4. The rules of thumb for predicting the oral availability and toxicity properties of the ligands studied were detailed in Table S2, and compounds IV and V meet the Lipinski, Veber, and Pfizer scales. Therefore, we can consider these ligands have potential for further research. I, II, and III exhibited high lipophilicity, and a higher TPSA, leading to insufficient candidates because these parameters indicate certain toxicological consequences.

## 3. Materials and Methods

### 3.1. Preparation of Protein

The apo protein structure of Mps1 (PDB Code: 6H3K) was downloaded from Protein Data Bank (http://www.pdb.org (accessed on 11 June 2021)), we found residues 674–685 and residues 698–708 were missing in the crystal, and residues 674–685 were in the active pocket. Therefore, we used the MODELLER 9.13v software package [23] to complete the residues and renamed the protein molecule to 6h3kmodeller. The protein structure was further analyzed by Ramachandran diagrams, the PROCHECK server was used to check the stereochemical quality of the protein structure, and the structure evaluation server ERRAT environment pro was used for analysis [26]. The ligands and water molecules were removed, and we used H++ server [27] to determine the protonation state of the protein at neutral pH, considering the effect of the protonation of the protein residues histidine on the calculations. We found that His645 and His796 were protonated at the Nδ and Nε positions; His581, His636, His639, His745, and His788 were protonated at the Nδ position; and His642 and His752 were protonated at the Nε position.

### 3.2. Molecular Docking

The crystal structure used for docking was a reasonably stable conformation obtained after pretreatment. In order to obtain a reasonable conformation of the 26 compounds, we drew all the compounds with Gaussian View [28], and used the B3LYP/6-31G method to obtain the most stable conformation. Afterward, the conformations with the lowest energy were selected for the following molecular docking. In order to obtain the initial structure of the complex of 26 compounds with Mps1, when docking by Autodock [29], all the residues of the active site were rigid. In the docking calculation, the Lamarckian genetic algorithm (LGA) [30] was set as the search algorithm; the number of cycles was 100; the number of search constellations was set to 100; the maximum number of energy evaluations was set to 2,500,000; the maximum number of generations was set to 27,000, and other parameters used default parameters. The definition of the 3D grid box was combined with the size of the pocket by using the Autogrid program. The x, y, and z axes were divided into 60 grid points. The center coordinates of the grid box were −1.67, −37.03, −7.378, and the grid point spacing was 0.375Å and used to define the size of the three-dimensional grid box. Verification of the molecular docking method was to verify the docking method used in this chapter through redocking. The original 6H3K ligand Bos172722 was docked back into the receptor protein according to the above method, and the docking result was superimposed and compared with the crystal structure 6H3K, and the reliability of the docking method was judged by the degree of overlap between the two structures. The root mean square deviation (RMSD) of the two Cartesian coordinates was usually used to measure success. If the RMSD of the docking pose was less than the threshold of 2Å, the docking was generally considered to be successful.

### 3.3. Molecular Dynamics Simulation

MD simulations can provide detailed information on structural changes at the atomic level and have been widely used in structural studies on protein molecules. In addition, MD simulations have the potential to provide a deeper understanding of Mps1 structure–function relationship than is available from experimental techniques such as X-ray crystallography.

In this article, the MD simulations for the complementary residues, the pretreated pure protein and four Mps1-inhibitor complexes were carried out using AMBER 16 software package and the ff14SB force field [24,31]. To keep the whole systems neutral, chloride ions (Cl^−^) were added using the t-Leap procedure of AMBER 16 based on a coulomb potential grid. Each system was then solvated with the TIP3P water model [32] in a truncated octahedron box with a 10 Å distance around the solute. The complex structures were fixed with a 500 kcal/molÅ^−2^ constraint, and the solvent and ions from the whole systems were submitted to 10,000 steps of steepest decent minimization followed by 5000 steps of conjugate gradient minimization. Next, the minimization was repeated for 5000 steps steepest decent minimization and 10,000 steps conjugate gradient conjugate gradient minimization without restraints. Subsequently, the systems were heated from 0 to 310 K in 500 ps by a Langevin dynamics with the collision frequency of 1 ps^−1^. Then, the systems went through 500 ps equilibrium MD simulations. Finally, a total of 200 ns MD simulation was performed for each system under NPT ensemble condition using periodic boundary conditions and particle-mesh Ewald (PME) for long range electrostatics [32,33,34,35,36]. Short-range interactions were cut off at 12 Å, and bonds involving hydrogen were held fixed using the SHAKE algorithm [37]. The time step was set to 2 fs. Data analysis was performed using the cptraj module of AmberTools16.

### 3.4. MM/GBSA Binding Free Energy Calculations

The MM/GBSA and MM/PBSA methods were considered to be two effective methods for estimating binding free energies. However, evaluating the performance of the MM/GBSA and MM/PBSA methods by calculating the binding affinity of ligands based on different molecular systems has found that the MM/GBSA [25] method can produce more attractive results. Therefore, in this article, the MM/GBSA method was used to estimate the binding free energy of Mps1 to A, BOS172722, C, D by the following equations [20,21]. The binding free energy was calculated by sampling the average value of 500 frames of conformations from the molecular dynamic’s trajectory in the last 10 ns every 20 ps. We can use the formula (1) to get MM/GBSA.

ΔG_bind_ = G_complex_ − (G_receptor_ + G_ligand_), (1)

G = E_MM_ + G_sol_ − TS,

E_MM_ = E_int_ + E_ele_ + E_vdw_,

G_sol_ = G_GB_ + G_SA_.

G_complex_, G_receptor_, G_ligand_ represent the free energy of complexes, proteins, and inhibitors, respectively. E_MM_, G_sol_, and TS, respectively, represent the molecular mechanics term, the solvent effect contribution term and the conformational entropy contribution term in the gas phase. E_MM_ can be obtained by the sum of electrostatic interaction energy (E_vdw_), van der Waals interaction energy (E_int_), and internal energy (E_ele_). Solvent effect (G_sol_) can be obtained by the addition of polar solvent effect (G_GB_) and non-polar solvent effect (G_SA_). G_GB_ can be obtained using the GB model, and G_SA_ can be obtained through surface tension and solvent-accessible surface area.

∆G_total_ = ΔE_vdw_ + ΔE_ele_ + ΔG_pol_ + ΔG_nopl_ (2)

Energy decomposition analysis can obtain information about important amino acid residues when Mps1 interacts with compounds, and compare the differences in the contribution of important residues in each system. We decomposed the total free energy of binding between molecules to all residues. As shown in formula (2), the binding free energy of each residual can be decomposed into van der Waals contribution (ΔE_vdw_), electrostatic contribution (ΔE_ele_), polar solvation energy (ΔG_pol_), and non-polar solvation energy (ΔG_nopl_).

### 3.5. Drug Design

LUDI is a fragment-based de novo drug design algorithm. According to our analysis, the core fragment of BOS172722 was selected as the starting point for drug design. Executed LUDI was added to new fragments in the template and it was ran on Discovery Studio 2.5 [38]. In the search, Lys529 was defined as the search center. The Linkage parameter was set to use all hydrogen atoms. The remaining parameters were the default settings. Density functional theory B3LYP/6-G in Gaussian09 software for molecular geometry optimization was used.

After obtaining the designed compound, we used Autodock to perform molecular docking to obtain the binding energy of the complex. We also used the SWISS-ADME [39] to evaluate the rationality, and finally obtained two desirable compounds.

## 4. Conclusions

In this article, the interaction mechanism between pyrido [3,4-*d*] pyrimidine, a new type of Mps1 inhibitors, and Mps1 was explored through the methods of molecular docking, molecular dynamics simulation, and energy calculation. In the previous literature [20,21], it was proposed that Gly605 can form a key hydrogen bond with inhibitors. In this study, we further proved the correctness of the above viewpoint. In addition, an important discovery of this study was that Lys529 can also form an important hydrogen bond with most inhibitors, which can further enhance the binding of Mps1 and inhibitors. This conclusion is reported for the first time. Second, we also confirmed that the shape of the substituent at the R_2_ position hypothesized in the literature was also important. When it is a “boat-shaped” structure, the inhibitor can be closely connected to the key hydrophobic pocket of Mps1. Analysis of binding free energies and hydrophobic interaction analysis revealed that Ile531, Val539, Cys604, Gly605, Leu654, Ile663, and Pro673 residues formed strong interactions with the inhibitors. The new insights obtained from this study can help to understand the binding modes of Mps1 inhibitors.

## Figures and Tables

**Table 1:** Docking results for 26 compounds.

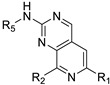					
**Inhibitors**				**Docked** **Energy** **(kcal** **/mol)**	**IC_50_** **(nM) [20,21]**
**Number**	**R_1_**	**R_2_**	**R_5_**		
33a	H	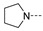	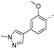	−8.49	330
33b	H	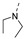	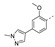	−7.98	1000
34a	H	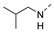	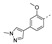	−8.48	370
34b	H	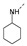	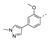	−8.53	130
34c	H	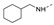	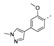	−7.99	1000
34d	H	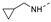	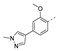	−8.51	280
34e	H	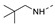	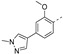	−8.57	44
34g	H	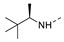	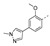	−8.47	420
34h	H	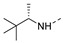	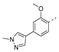	−8.60	20
14(A)	H	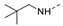	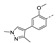	−7.83	2500
15	H	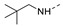	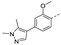	−8.32	79
16	H	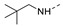	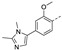	−8.96	8.5
17	H	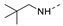	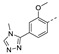	−9.01	6.8
34	Me	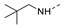	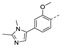	−8.49	9.5
35	Me	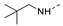	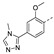	−8.48	13
36(BOS)	Me	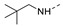	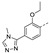	−8.51	11
37	Me	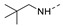	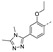	−8.48	12
38	Me	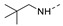	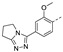	−8.47	19
39	Me	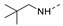	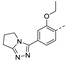	−8.45	38
40	Me	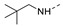	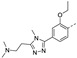	−8.50	12
41	Me	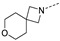	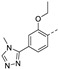	−8.51	11
42	Me	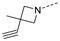	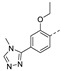	−8.47	20
46	Me	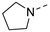	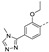	−8.34	83
47(C)	Me	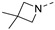	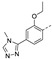	−8.91	8.9
48(D)	Me	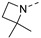	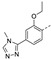	−8.16	400
45	Me	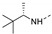	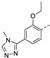	−9.12	4.7

**Table 2:** The predicted binding free energy and the individual energy components of the binding for the studied molecules (kcal/mol).

Inhibitor	A	BOS172722	C	D
ΔE_vdW_	−58.04 +/− 2.96	−60.99+/−2.87	−61.60+/−3.78	−59.12+/−3.69
ΔE_ele_	−5.45 +/− 3.45	−29.34+/− 4.58	−26.46+/−7.54	−27.13+/−6.97
ΔG_GB_	25.83+/−3.27	45.59+/− 5.14	41.21+/−7.17	43.56+/−7.71
ΔG_SA_	−6.80+/−0.30	−6.84+/−0.29	−6.70+/−0.23	−6.81+/−0.34
ΔG_pol_^a^	20.38	16.25	14.75	16.43
ΔG_nonp_^b^	−64.84	−67.83	−68.30	−65.93
ΔG_MM-GB/SA_^c^	−44.47+/−3.29	−51.58+/−2.93	−53.55+/−4.17	−49.50+/−3.54
−TΔS	15.42+/− 4.56	13.12+/−3.19	13.60+/−3.28	14.39+/−4.59
ΔG_bind_^d^	−29.07+/−3.37	−38.46+/−2.97	−39.95+/−3.52	−35.11+/−3.25
IC_50_Mps1(nM)	2500	11	8.9	400

^a^ ΔG_pol_ = ΔE_ele_ + ΔG_GB_; ^b^ ΔG_nonp_ = ΔE_vdW_ + ΔG_SA;_ ^c^ ΔG_MM-GB/SA_ = ΔE_ele_ + ΔG_GB_ + ΔE_vdW_ + ΔG_SA_; ^d^ ΔG_bind_ = ΔG_MM-GB/SA_ − TΔS.

**Table 3:** Analysis of hydrogen bond interactions.

Inhibitors	Donor and Acceptor	Average Distance (Å)	Average Angle (°)	Occupation Rate (%)
A	Gly605-N-H…A-N16	2.93	162.01	88.64
	Gly605-N-H…A-H4	2.92	142.52	3.11
	A-N10-H…Gly605-O	2.86	147.44	8.25
B	Gly605-niN-H…BOS-N6	2.93	155.88	33.12
	Lys529-NZ-H…BOS-N8	2.92	154.73	39.61
	Lys529-NZ-H…BOS-N9	2.91	152.81	27.23
C	Gly605-N-H…C-N22	2.92	156.03	47.43
	Lys529-NZ-H…C-N13	2.91	154.79	28.94
	Lys529-NZ-H…C-N14	2.92	152.70	22.37
D	Gly605-N-H…D-N22	2.93	156.45	41.20
	Lys529-NZ-H…D-N13	2.92	155.27	37.20
	Lys529-NZ-H…D-N14	2.91	152.53	18.35

**Table 4:** Ligand efficiency parameters calculated and absorption, distribution, metabolism, excretion, and toxicity (ADME-Tox) properties prediction for the five new compounds studied.

	Docked Energy	cLogP	HBA	HBD	TPSA	RB	Lipinski Rules/Veber Rules/Pfizer 3/75 Rules
I	−8.92	2.87	7	2	110.63	8	Yes/Yes/No
II	−8.66	4.81	5	2	85.2	8	No/Yes/No
III	−8.62	4.78	5	2	81.19	8	No/Yes/No
IV	−8.86	2.31	6	1	70.38	6	Yes/Yes/Yes
V	−8.89	2.87	7	1	73.87	8	Yes/Yes/Yes

## Data Availability

The data presented in this study are available in supplementary material.

## Supplementary Materials

The following are available online, Figure S1: Ramachandran diagram of the 3D structure of Mps1 before optimization, Table S1: Residue decomposition energy, Table S2: Empirical rules for predicting the oral availability and toxicity properties of the studied ligands.

## Sample Availability

Samples of the compounds are not available from the authors.
