# Supplementary material for: Studies of Interaction Mechanism between Pyrido [3,4-d] Pyrimidine Inhibitors and Mps1
Source: Molecules. 2021 Aug 21;26(16):5075. doi: 10.3390/molecules26165075 (PMC8401005; doi:10.3390/molecules26165075)
Supplement: Supplementary file 1 [file molecules-26-05075-s001.zip › molecules-1276512-supplementary.pdf]

Supplementary Materials: Studies of Interaction Mechanism Between pyrido[3,4-d] pyrimidine inhibitors and Mps1

Cheng Xing1, Xiaoping Zhou1, Chengjuan Chen1, Wei Sun1, Qingchuan Zheng2, Di Liang1,*

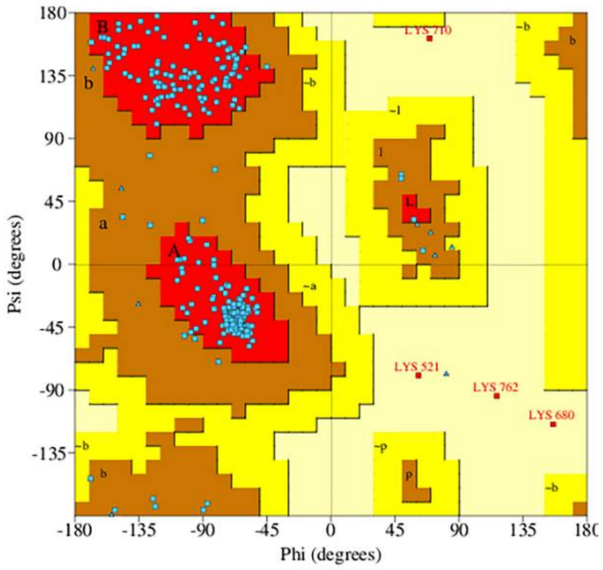

Figure S1. Ramachandran diagram of the 3D structure of Mps1 before optimization.

TableS1 Residue decomposition energy

	Residues	Vdw	nonp	ele	Pol	total
	Ile531	-3.58	-0.53	0.82	-0.47	-3.80
	Val539	-1.67	-0.18	-0.01	-0.19	-2.10
	Ala551	-0.62	-0.12	0.16	-0.05	-0.60
	Ile586	-0.67	0.01	0.03	-0.04	-0.70
	Met602	-0.75	-0.03	0.03	-0.07	-0.80
	Cys604	-1.25	-0.88	0.56	-0.04	-1.60
	Gly605	-1.16	-1.87	2.14	-0.11	-1.00
	Ans606	-1.52	-0.38	1.42	-0.17	-0.60
Mps1-A	Ile607	-1.90	0.29	-0.20	-0.18	-2.00
	Ser611	-0.50	-0.19	0.28	-0.08	-0.50
	Leu654	-2.16	-0.28	0.09	-0.21	-2.60
	Ile663	-1.38	-0.03	0.07	-0.27	-1.60
	Pro673	-1.55	-0.07	0.07	-0.21	-1.80

Mps1-B	Lys529	-0.40	-9.49	9.38	-0.19	-0.70
	Ile531	-3.20	-0.52	0.87	-0.46	-3.30
	Va639	-1.75	-0.05	-0.01	-0.18	-2.00
	Ala551	-0.58	-0.06	0.17	-0.05	-0.50
	Ile586	-0.71	-0.01	0.06	-0.05	-0.70
	Met602	-1.39	-0.08	0.04	-0.08	-1.50
	Cys604	-1.37	-1.44	0.89	-0.06	-2.00
	Gly605	-2.00	-2.43	2.74	-0.17	-1.80
	Asn606	-1.92	-0.83	2.14	-0.25	-0.90
	Ile607	-1.04	0.548	-0.20	-0.06	-0.70
	Leu654	-2.43	-0.29	0.18	-0.23	-2.80
	Ile663	-1.77	-0.10	-0.28	-0.01	-1.80
	Met671	-1.33	-0.19	0.94	-0.19	-0.80
	Pro673	-1.20	0.102	-0.10	-0.18	-1.20
Mps1-C	Ile531	-3.18	-0.6	0.85	-0.43	-3.36
	Lys529	-0.46	-7.91	8.04	4.58	-0.54
	Val639	-2.10	-0.13	-0.01	-0.19	-2.40
	Ala551	-0.64	-0.06	0.13	-0.04	-0.61
	Ile586	-0.78	-0.01	0.06	-0.06	-0.79
	Met602	-2.00	0.075	-0.01	-0.08	-2.00
	Cys604	-1.27-	-1.17	0.7	-0.05	-1.79
	Gly605	-1.74	-2.68	2.73	-0.14	-1.83
	Asn606	-1.96	-0.43	1.58	-0.25	-1.06
	Ile607	-1.18	0.431	-0.2	-0.08	-1.02
	Leu654	-2.39	-0.333	0.14	-0.23	-2.82
	Ile663	-1.52	-0.05	0.12	-0.2	-1.74
	Met671	-1.57	-0.19	0.44	-0.18	-1.50
	Pro673	-1.6	0.39	-0.3	-0.19	-1.70
	Ile531	-2.94	-0.65	0.987	-0.35	-2.96
	Val539	-1.93	0.03	-0.151	0.006	-2.04
	Lys529	-0.418	-8.28	8.231	-0.186	-0.66
	Ala551	-0.69	-0.05	0.149	-0.193	-2.30

	Ile586	-0.75	-0.01	0.04	-0.04	-0.74
Mps1-D	Met602	-1.9	-0.01	0.1	-0.07	-1.8
	Cys604	-1.23	-1.16	0.706	-0.05	-1.73
	Gly605	-1.84	-2.77	2.92	-0.15	-1.83
	Asn606	-2.22	-0.45	1.62	-0.27	-1.33
	Ile607	-1.27	0.4	-0.124	-0.07	-1.07
	Leu654	-2.18	-0.32	0.2	-0.2	-2.5
	Ile663	-1.69	-0.26	0.27	-0.25	-1.92
	Met671	-0.79	-0.04	0.33	-0.11	-0.61
	Pro673	-0.87	0.91	-0.17	-0.17	-0.30

Table S2. Empirical rules for predicting the oral availability and toxicity properties of the studied ligands

Properties	Lipinski Rules	Veber Rules	Pfizer 3/75 Rules
MW	≤500	-	-
cLogP	≤5	-	≤3
HBA	≤10	-	-
HBD	≤5	-	-
TPSA	-	≤140	≤75
RB	-	≤10	-

MW: Molecular weight; LogP: Octanol/water partition coefficient; HBA: Hydrogen bond acceptor; HBD:Hydrogen bond donor; TPSA: Topological polar surface area; RB: Rotatable bond count.
